# What Determines Pig Farmers’ Epidemic Coping Behaviors: A Qualitative Analysis of Endemically Infected Areas in Relation to African Swine Fever

**DOI:** 10.3390/vetsci8110266

**Published:** 2021-11-05

**Authors:** Bo Xu, Lijuan Zhou, Chengmei Qiu, Yanling Li, Wei Zhang

**Affiliations:** 1 College of Public Administration and Law, Hunan Agricultural University, Changsha 410128, China; xb1244118@163.com (B.X.); zlj2016jane@hunau.edu.cn (L.Z.); liyanling1964@hunau.edu.cn (Y.L.); 2 School of Medicine and Health Management, Huazhong University of Science and Technology, Wuhan 430030, China; weizhanghust@hust.edu.cn

**Keywords:** pig farmers, epidemic coping behavior, African swine fever, epidemic prevention and control

## Abstract

An animal epidemic is a big threat for economic development that may seriously disturb the breeding industry and people’s normal life. The most effective approach so far for epidemic control is biosecurity, zoning, culling animals exposed, and other relevant measures, which highly demands the cooperation of farmers in epidemic areas. However, an uncooperative phenomenon among individual farmers facing an epidemic has been recorded for a long time and includes unwilling to report the epidemic and selling infected pork. It is important to unravel the determinants of farmers’ coping behaviors during an animal epidemic outbreak and use corresponding strategies to reduce farmers’ inappropriate behaviors. Taking African Swine Fever (ASF) crisis as an example, this study aimed to reveal the determinants and underlying mechanism of pig farmers’ coping behaviors. We adopted qualitative interviews with 45 pig farmers across four endemically infected areas in Hunan provinces, and the data collected were subjected to a grounded theory analysis. Our results showed that emergency response, information sources, and information channels jointly affected pig farmers’ epidemic risk perception and their perception of coping behaviors. Meanwhile, both the characteristics of the government and pig farmers moderated this affect. Consequently, by processing information through either a heuristic or an analytical path, pig farmers’ behavioral intention was transformed into actual coping behaviors. Our study emphasizes the value of sufficient risk communication, proper compensation policies, and strong public trust in the government for improving the farmers’ participation in the epidemic response. Theoretical and practical implications to animal epidemic prevention and control are provided.

## 1. Introduction

### 1.1. African Swine Fever in China

African Swine Fever (ASF) is an acute, hemorrhagic, and severe infectious disease caused by the African swine fever virus infecting domestic pigs and wild boars [1], and leads to almost 100% mortality [2]. It was first recorded in 1921 when it emerged as an acute haemorrhagic fever in domestic pigs in East Africa [3], and seldom spread out of African countries before the 1950s [4]. The first ASF case outside Africa was reported in Europe in 1957, and since then, it has gradually spread to other continents [5]. The world organization for animal health (OIE) lists it as a legally reported animal epidemic. The first reported ASF outbreak in Asia was in North-East China (Shenyang City, Liaoning province), in August 2018 [5,6]. Since then, it has been found across the nation. Although much effort has been dedicated to the development of an ASF vaccine, there is still no significant progress in the field [7,8]. Since no recognized treatment for ASF exists, the most commonly adopted strategies for disease prevention and control are primarily on biosecurity, early detection and reporting, movement restrictions and zoning, and culling animals exposed [5,8].

Since the first China ASF case reported in August 2018, all provinces have experienced ASF. There were 99 ASF outbreaks reported in 2018, stamping out over 800,000 pigs. In 2019, 63 ASF outbreaks were reported, with 390,000 pigs slaughtered, and 19 ASF outbreaks with 13,500 pigs slaughtered. By 11 July 2021, ASF outbreaks were reported and 2216 pigs were slaughtered. The outbreak of animal epidemic caused great economic losses, jeopardized the development of the breeding industry, and seriously disturbed people’s normal production and life [9]. Since the most effective approach to control the spread of ASF is quarantine, isolation, culling, harmless treatment, disinfection, and other measures, the pig farmers’ participation and their cooperation in epidemic affected areas is of great importance. According to the latest *Emergency Implementation Plan for African Swine Fever Epidemic* issued by the Chinese Ministry of Agriculture and Rural Affairs, quarantining infected areas require prohibiting the entry and exit of susceptible animals and products in relation, and setting up temporary inspection and disinfection stations. Harmless treatment highlights to the process of treating diseased and dead animals and related animal products by physical and chemical methods to eliminate the pathogens they carry. Extant studies revealed that pig farmers’ coping behaviors were not always complying with government interventions, for example, not cooperating with preventive measures, unwilling to report the epidemic, and selling infected pork in an animal epidemic [10,11]. Therefore, it is of great significance to explore the mechanism of farmers’ coping behaviors in an outbreak of animal epidemic in order to formulate targeted strategies.

### 1.2. Explanations for Pig Farmer’s Coping Behavior in Epidemic

Coping behavior refers to individuals’ management and adaptation towards specific stressors through psychological and operational coping measures [12]. Coping behaviors of pig farmers in ASF epidemics in this study refer to their psychological and behavioral coping strategies towards ASF. In China, since pig farmers’ prevention and control towards ASF are mainly guided by government response and governmental epidemic prevention policies, pig farmers’ coping behaviors are pig farmers’ compliance with government epidemic prevention policies and their psychological intention at the individual level.

Efforts on pig farmers’ coping behaviors and their influencing factors had been well established. In general, they can be divided into two strings, one of economic assumption and the other of psychological assumption. For the economic assumption, farmers’ coping behaviors are based on their calculations of the gain and loss from an economic perspective [13]. In this sense, pig farmers’ adoption of epidemic preventive measures is largely determined by their comparisons of the cost in conducting preventive measures and the effectiveness of these measures for loss avoidance [14,15]. From this perspective, the economic incentive is believed as an effective tool to influence the farmers’ willingness to conduct preventive measures [16,17]. For example, a research into pig farmers in rural Uganda found that the implementation of biosafety measures did reduce ASF outbreak, but resulted in a 6.3% reduction in pig farmers’ annual profit rate [18]. Another research in Uganda also revealed that the only understanding of control measures does not guarantee pig farmers’ adoption of those measures, because they rely heavily on their pigs raised to reduce poverty [19]. Studies in Netherlands and Belgium also confirm the crucial role of the farmers’ perception of benefits in their adoption of preventive measures [20,21]. However, some studies suggested that a government economic compensation policy might have adverse incentives. Researchers pointed out that a substitution relationship may exist between the government’s compensation level and the farmers’ efforts in implementing epidemic prevention [22]. Some explained that the government epidemic prevention and control measures may be effective in stopping the transmission, but may also distort the market signal and reduce farmers’ individual epidemic prevention motivations [23].

Another string of research featured on psychology, and it was perceived that farmers’ coping behaviors are the result of multiple psychological factors, such as individual risk perception, trust, responsibility, culture, and knowledge [11,24,25]. For example, Claireguinat et al. [11] revealed that pig farmers’ active reporting activity was largely depended on their familiarity with clinical symptoms of ASF and their cognition of the complexity of reporting procedures. Further, Covello et al. [25] evidenced that individuals’ perception of animal disease risk significantly affects pig farmers’ coping behaviors by examining river virus epidemics in New York City. According to these researchers, they emphasize risk communication strategies to guide farmers from a rational risk perception. The strategies mainly strengthen epidemic information provision on disease, probability, and government control measures. Nantima Noelina et al. [26] found that government support helped to improve farmers’ risk perception and adopt biosafety measures in villages in Uganda and Kenya. However, recent studies also showed that epidemic knowledge might not affect farmers’ coping behaviors. For example, farmers may still sell infected pork anyway, although they understand its harm to human health, for a low risk of legal punishment in Madagascar [27]. Another study employed a randomized controlled trial (RCT) to evaluate the effect of participatory training on the biological safety knowledge about ASF, attitude, and practices of pig farmers in Uganda. The results showed that biosafety training had a significant impact on farmers’ knowledge acquisition after 12 months, but farmers’ attitudes and behaviors changed only slightly after 12 and 28 months [19]. In other words, knowledge is not a deciding factor in biosafety interventions.

Researchers in China have identified many factors influencing farmers’ coping behavior in epidemic, and they have distinguished factors for specific preventive behaviors. For example, the epidemic reporting behavior is associated with education level, epidemic prevention awareness, understanding of epidemic disease [28], whether to participate in training, compensation for loss of culling, proportion of income from breeding in family income, and trust in grass-roots government [29]. On the other hand, culling behaviors correlate with age, income, education level, cognition of epidemic disease, trust in local government, and compensation satisfaction, and the main factors impacting the pig farmers’ handling of affected pigs include breeding scale, proportion of breeding income in total household income, accessibility of disposal sites, severity of government punishment, farmers’ satisfaction of compensation, and farmers’ loss aversion to detention [30,31]. However, few studies have explored the mechanism of how these factors affect farmers’ coping behaviors. This paper adopted a grounded theory approach to analyze the formation mechanism of farmers’ coping behaviors with 45 pig farmers in the ASF epidemic affected areas. This research can shed lights on policy-making for animal epidemic emergency management.

## 2. Materials and Method

### 2.1. Data Collection

Since August 2018, China has experienced a number of ASF outbreaks. The Ministry of Agriculture and Rural Affairs released the outbreak information in four infected areas in Hunan province, including Taojiang County (Yiyang City), Taoyuan County (Changde City), Lianyuan City (Loudi City), Hecheng District (Huaihua City), respectively on October 23, November 8, and November 21, 2018. After the ASF outbreak was detected, the local emergency response mechanism was activated correspondingly, mainly targeting the pig breeding industry. Focusing on these four areas, this study adopted qualitative interviews with pig farmers right after the release, ranging from November to December 2018. Selecting ASF affected areas in Hunan province was mainly done out of the following two considerations. Firstly, Hunan is a major pig-raising province. According to the data in *China Statistical Yearbook*, the size of pig slaughtered in Hunan ranked second nationwide and the size of live pig stock ranked third in 2019. Secondly, the locality of Hunan and their pig-raising custom make it a representative sample for exploring pig farmers’ epidemic coping behaviors. Hunan is located in central China, and before the ASF outbreak, cross-regional transfers of live pigs and their products were frequent. Besides, the free-range pig rearing method is quite common in rural Hunan province, and pig farmers share a tradition of feeding pigs with swill. All these factors make ASF prevention and control in Hunan be distinguished from other areas, but also representative.

As for the recruiting criteria, firstly, we selected one village affected the most from each of the four areas. Then, we select approximately 10 pig farmers from each of the four selected villages. The final number of the participants was determined by the balance of pig farmers’ gender, breeding size, education background, which were likely to influence their coping behaviors. If a balanced sample was reached, the recruitment in the village would stop. Finally, 45 valid interviews were obtained with 11 from Taojiang County, 10 from Taoyuan County, 15 from Lianyuan City, and 9 from Hecheng District. All the pig farmers must have had already cullied their pigs, which made them perfect participants in this study. Among them, 32 pig farmers’ breeding size was below 30 heads, taking up 71.1%, which is close to the overall situation of China’s pig farming industry. The interview consisted of two sections after a brief introduction of the study. In the first section, we collected background information of the pig farmers, such as their gender, education, pig breeding experience, and current breeding size. In the second section, we asked their risk perception, attitude towards government measures, their coping behaviors, and underlying reasons. (Please refer the Appendix A for the details.) We had three interview teams and each team had three trained interviewers with one expert in animal epidemic prevention and control from Hunan Agricultural University at least. We adopted a semi-structured interview approach to obtain information, because of its flexibility for adjustment according to individual interviewee’s response to make in-depth explanation on how the person experienced the situation. Each interviewee had enough time to describe and express their views and emotions on the ASF outbreak. The interview team made detailed on-site records and paid sufficient attention to their mood, intonation, and facial expression to understand their real feelings [32]. Each participant was interviewed for at least 30 min.

### 2.2. Analytical Methods

Grounded theory methodology is the most influential approach for researchers to handle qualitative materials, and it has been widely used across multiple disciplines [33]. Through constant comparative analysis with the materials, vigorous coding procedures and reflections, the researchers are expected to form a new perspective to understand the emerging problems or develop new theories explaining them. Although the attitude and coping behaviors of pig farmers in epidemic situations has been studied extensively [10,11,24], little attention was devoted to the explanation of their coping behaviors. To this aim, this study adopted a grounded theory approach to reveal the determinants and underlying mechanism of pig farmers’ coping behaviors in epidemic situations.

Following the practical guide on data saturation [34], we estimated the minimal interviews for coding saturation and meaningful saturation by taking our research aim into consideration. Finally, we randomly selected 30 interviews materials for an initial model construction, and left 15 for theoretical saturation check. As suggested, all the recordings were transcribed and subjected to a three-step coding mechanism [33]. Indeed, using a random selection of 2/3 of the total interviews for constructing a theoretical model and the remaining 1/3 for data saturation tests is a common adopted approach in grounded theory analysis. We coded the 45 interviews, and randomly selected 30 interviews for model construction, and used the remaining 15 for theoretical saturation check [35]. Specifically, the coding process including open coding, axial coding, and selective coding, and was assisted by NVivo 12. Subsequently, the determinants and underlying mechanism of farmer’s coping behaviors were proposed.

## 3. Results

Of the 45 participants, 31 were male and 14 were female. Among them, 21 had junior high school education or lower, 18 people had senior high school education, and 6 people had junior college education. As for the breeding size, 32 raised less than 30 pigs, 11 raised a number of pigs ranging from 30 to 100, and 2 raised more than 100 pigs. Detailed information is presented in Table 1.

### 3.1. Open Coding

In the open coding stage, the major aim is to identify import content and conceptualize the content correspondingly. We carefully examined each interview material sentence by sentence, and extracted words with important meanings for conceptualization. Then, we tried to aggregate similar concepts together for an initial classification. Finally, 76 concepts and 27 preliminary categories were obtained. See Table 2 for details.

### 3.2. Axial Coding

The goal of axial coding is to identify connections and relationships between concepts and develop main categories from identified preliminary categories [36]. Interpretive and conceptual patterns and relationships can be identified by looking for recurring phenomena, events, actions, and interactions and placing them [37]. For example, all behavioral perceptions were related to the main category *Coping Behavior Perception*, which included the preliminary categories such as *Behavioral Cost* and *Behavioral Efficacy*. Twenty-seven initial categories and 10 main categories were developed. See Table 3 for details.

### 3.3. Selective Coding

By comparing all the initial categories and main categories, the core categories centered on the determinants and underlying mechanism for farmers’ response behavior, which coincides with the theoretical framework of Cognition–Evaluation–Reaction in decision-making for preventive behaviors. The typical story line is in the ASF epidemic scenario; pig farmers go through a series of evaluation process including risk perception, cost-benefit calculation of personal preventive activities, and decision-making and then take corresponding responsive behaviors. Among them, the epidemic scenario is a decisive factor for farmers’ epidemic coping behaviors. Farmers perceive the risk from the epidemic scenario and realize the necessity of epidemic prevention and control from the emergency response of the government and others, which initiate an evaluation process. The evolution is of great significance for the final decision-making and actual preventive behaviors of pig farmers, and their variations in evaluation of ASF prevention and control lead to variations in actual behaviors in coping ASF. Differences in the process of assessing farmers’ outbreak response behaviors lead to differences in response behavior choices.

**Epidemic scenario**. Epidemic scenario is defined as the social response triggered by the epidemic, information dissemination, and its impact on farmers. The government and public response quickly towards the epidemic. In the epidemic scenario, various ASF information served as stimulus, as well as government efforts and their own condition for pig farmers to form their perception towards ASF. Therefore, we categorized the five main categories including emergency response, information sources, information channels, governmental epidemic prevention characteristics, and farmer characteristics into the epidemic scenario.

**Individual decision-making process.** Individual decision-making process refers to the farmers’ evaluation of the epidemic risk and their own preventive behaviors. After closely observing the government response towards ASF, pig farmers develop their initial personal understanding of the ASF and its impact on their breeding industry, for example, the probability of epidemic occurrence and the severity of consequences. Based on their initial calculation, they develop their risk perception towards epidemic and personal coping behaviors, which help them to make a decision. In this sense, we have classified three main categories, namely epidemic risk perception, perception of personal preventive behaviors, and behavioral decision into evaluation of epidemic prevention and control.

**Actual coping behavior**. Actual coping behaviors are final behaviors implemented by pig farmers after their decisions on individual epidemic coping behaviors. After a comprehensive evaluation of government efforts on epidemic prevention and control, pig farmers make their final decision of epidemic response on the personal level, i.e., whether to follow the government guidelines or not, and if followed, to what extent. Generally, we have summarized two main coping strategies, one of problem-orientedness and the other of emotion-oritentedness. Problem-oriented coping behaviors refer to problem-solving and seeking instrumental social support to address the problem, typical behaviors are culling, biosecurity, reporting, and handling of dead pigs. Emotional-oriented coping behaviors are connected to negative emotion relief, and typical behaviors are emotional vetting and seeking emotional support.

### 3.4. Data Saturation Check

The remaining 15 interview materials were subjected to the same coding process to assess whether a new concept emerged. Data Saturation Check shows the main line of formation mechanism of pig farmers’ epidemic coping behaviors, and no new categories have emerged, and no new relationships have been found between various categories. In this sense, the core category of the model can be considered to have reached saturation.

## 4. Discussion

**Figure 1:**
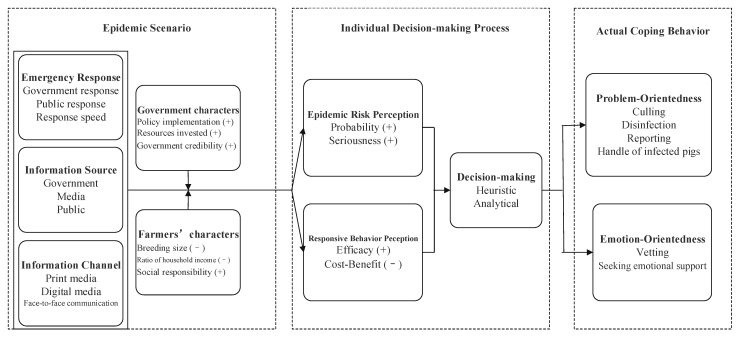
Formation mechanism of pig farmers’ epidemic response behavior. Note: + refers to a positive impact, while—refers to a negative impact.

The formation mechanism of farmers’ epidemic response behavior is shown in Figure 1. The three relationship categories of epidemic situation, evaluation of epidemic prevention and control, and epidemic response behavior reveal the formation process of the farmers’ epidemic response behavior. The specific explanation is as follows.

### 4.1. Impacts of Epidemic Situation on Pig Farmer’s Evaluation

Firstly, emergency response, information sources, and information channels have had an important impact on the pig farmers’ evaluation of current epidemic response behaviors. First, the government and public emergency response and various informations are the sources for pig farmers to develop their risk perception of the outbreak. When ASF occurred in Hunan for the first time, pig farmers did not understand the origin, transmission channels, and epidemic prevention policies of ASF, resulting in a low risk perception during the early stage of the epidemic. A pig farmer in Taoyuan County has confirmed this as follows:

*I raised pigs for decades. I have never heard of ASF. Should not ASF come from African, and why it was found here... We use to feed pigs with swill for quite a long time and nothing bad happen. The swill is full of nutrition for pigs to grow up. Pigs fed with swill is more delicious than those pigs raised in big farms. People in our place would pay more money for those pigs fed with swill rather than buying pork from those pigs fed with fodder.*

(TY09)

The local government quickly initiated emergency response plans, and took emergency measures, i.e., quarantine, culling, and disinfection, official announcement of epidemic information, and indoor epidemic preventive information dissemination, which helped pig farmers develop a rational risk perception. For example, a pig farmer stated that:

*The staff in the town comes to all the households raising pigs twice a day to disinfect the pigpens. …… I heard they were saying that feeding pigs with swill might lead to pigs be infected with ASF... According to them (local staff in the village), ASF does not infect people.*

(LY04)

*Local government has set up temporary checkpoints on the way, and they disinfect people and cars passing by our village. It is said that they checked cars to make sure they would not carry any raw meat. I think the government has attached great attention on this, and this means that this infectious disease is very serious. How to say that, but I think since the government has taken it seriously, the problem will not be solved soon.*

(TY07)

Second, there are variations in the impact of information from different information channels on the risk perception of pig farmers. Compared with the information from the government, information from neighbors or unofficial media outlets can easily lead farmers to misunderstand the nature of the epidemic, leading to twisted risk perception. For example, some farmers have heard rumors on the social media that ASF can also infect chickens and ducks. Some believed that after culling the pigs, the government would also take measures to cull the chickens and ducks. To prevent further loss of the chickens and ducks, they slaughtered them and kept in the refrigerator. As one pig farmer reflected:

*Everyone in my WeChat group talks about this. Some said that ASF only infects pigs, while another group of people reposted message stating that it also infect other domestic animals, such as cattle, sheep, chickens and ducks. Those chickens and ducks are not infected, and why can’t we eat them? What a pity to have all the pigs buried! People around me have killed all the chickens and ducks and put them in the refrigerator, and I plan to kill my chickens and put them in the refrigerator too.*

(HC01)

Secondly, the government’s epidemic prevention features have a moderating effect on pig farmers’ evaluation of epidemic response behavior. The characters of government epidemic efforts in prevention and control highlight the implementation of the ASF prevention and control policy by the grassroots government, their investment of resources, and government credibility. The grassroots government has invested many labor and resources to implement the epidemic prevention policy, and this is likely to increase pig farmers’ perception of cost against epidemic prevention policies. For example:

*This ASF has a great impact in our life. Local government have dispatched to disinfect from house to house and conduct inspection at various locations. It is very strict. A majority of the people will cooperate with the policy, and only few people will not cooperate. After the explanation of local staff, almost all the people can understand and follow it. The local government spares no efforts, and two staff at the temporary station was given a warning punishment since they left without notice. At this very moment, for sure the government will definitely punish any violators severely anyone who hinders the prevention and control of the epidemic. No one dares to sell pork infected.*

(TY02)

On the other hand, the credibility of the government adjusts the farmers’ perception of epidemic preventive costs. The lower the government credibility, the higher the farmers’ perception of the cost of epidemic prevention. For example, one pig farmer expressed his concern as follows:

*We have experienced that the government compensation failed to allocate to us on time. The provincial government, city government and county government will take our money one by one. Although local staff had confirmed that we could get the money directly in our personal account, I still not that sure. We want to know how about this time; will we get our compensation on time? It is not refund if we cannot get the money. If the compensation were not paid, our farmers would suffer a great more loss.*

(LY05)

Thirdly, the characters of farmers have a moderating effect on farmers’ evaluation of epidemic response and control. Their characters mainly include a breeding scale, the contribution of breeding for family income, and the sense of social responsibility. First, the scale of farming and the contribution of breeding for family income are closely connected to their perceived cost of coping behaviors. On one side, the larger the scale of breeding, especially the larger the ratio of large pigs, the greater the loss pig farmers suffer due to culling. On the other side, the larger the contribution of breeding to the household income, the greater economic costs, i.e., breeding costs and profit losses, as well as time cost and reemployment cost farmers bear, excluding their cost for epidemic prevention and control. Therefore, the larger the breeding scale and the higher the contribution of breeding to the household income, the higher the farmers’ perception of the cost for epidemic prevention.

*“I have 37 pigs all culled. Among them, two were breeding pigs, around 15 pigs (weighted) more than 300 kg, 10 pigs (weighted) 200 kg, and the rest are piglets. Now the pigs are gone, and we have several hundred kilograms fodder. I have planted several acres of sweet potatoes, and I don’t know how to deal with it. To be honest, I don’t want to slaughter my pigs* *. Although they said that we could get compensation of 1200 yuan per head, I don’t know when we can get it. My wife and I raised pigs at home for a living for six or seven years. Pig raising is the entire income of our family. Since we have not any household income, we are so worried. You know, it is not easy to find something else to do for us in such a short time.*

(LY09)

In addition, farmers’ sense of social responsibility adjusts their perception of the benefits of epidemic prevention behavior. It is proved that in a public health crisis, the goodwill and sense of responsibility of the human nature will be aroused instantly. The moral sense of putting human life first and the sense of responsibility to face disasters together urge farmers to make behaviors conducive to epidemic prevention and control. The theory of Schwartz’s activation of norms [38], which means that when an individual realizes that failure to perform pro-social behavior will cause adverse consequences to others and that he is responsible for these adverse consequences, the individual’s sense of moral responsibility is activated, is verified. For example,

*“...I am a farmer. Although I don’t have much knowledge, I know that this disease is very contagious. It is a natural disaster. There is no other way. It is everyone’s responsibility to prevent and control the disease. Individuals who suffer some losses can only complain their bad luck. Whatever the government say, I will follow them, and hope that farmers in other places will not suffer losses (like us).*

(TJ02)

### 4.2. Decision-Making in an Epidemic by a Comprehensive Evaluation

Firstly, the epidemic risk perception refers to pig farmers’ perception of the probability of ASF occurrence and the severity of its consequences. The correlation between risk perception and corresponding coping behaviors have been tested theoretically and empirically. Our research has once again confirmed this correlation, i.e., the perception of epidemic risk affects the choice of epidemic response behavior. When farmers perceived a high probability of ASF occurrence and devastating consequences, they are more likely to adopt an active problem-orientedness approach in responding to the epidemic to minimize potential risks. For example,

*ASF is very contagious and has a high fatality rate. There is no medicine to cure it. I heard that pigs infected with this disease would undoubtedly die. If we do not report to the government on time, our pigs as well as other pigs would all die.*

(LY13)

Secondly, coping behavior perception refers to farmers’ perception of the effectiveness and cost of the epidemic response behavior they intend to adopt. Among them, behavioral effectiveness includes two aspects: farmers’ subjective assessment of their own ability to prevent epidemics, and the evaluation of their personal epidemic prevention behaviors on the overall epidemic prevention effect. Behavior costs include money cost, time cost, energy cost, and reemployment cost. If farmers have a higher benefit perception and lower cost perception towards the measures they intend to adopt, they are more likely to adopt corresponding response behaviors. For example,

*I will never slaughter my pigs privately at this time. You think, it is a very moment and the government attached such great attention. If caught, one would be taken to the police station, were fined, and may be laughed by your neighbors. No matter how much money I lose, it does not matter. If someone ate infected pork and got sick, it is a big deal. I will definitely not do that kind of thing.... Selling infected pork is very, very bad, I will definitely report it if I found out... Local staff in our village offer free disinfectant, and teach us how to use it and told us to disinfect pigpens twice a day inside and out. It is easy to sterilize inside and outside the pigpen twice a day. Doing this is both good for myself and others. We do not have to pay anything, and it would not take much time. However, it is good for everybody. I have sterilized (pigpens) as required every day since they told me to*.

(TJ10)

Thirdly, there are two pathways for farmers to make their behavioral decision: heuristic path and analytical path. The heuristic path is an approach relying on intuition to make decisions quickly and take corresponding actions. For instance, one farmer said:

*Government has its own reason for doing this. I will do whatever the government people required.*.

(LY06)

On the other hand, the analytical path is the active search for relevant information, process it, and make decisions after calculating the benefits and costs. For instance,

*I asked my son to check it. The policy stipulates that each pig culled can be compensated 1200 yuan. I think, as long as the government has compensated our loss, we have no reason to complain.*

(TJ05)

*I asked my neighbors, and all of them are willing to cooperate with the government* *. I think that I will cooperate too. I don’t want people to say that I am a selfish person or something like that.*

(LY11)

Analytical behavioral decision-making is a complex process. It is likely to be affected by government emergency management policies, but also individuals’ avoidance of economic losses, as well as face and social pressure. As one farmer responded to why he took these actions:

*Government staff and village cadres came to tell us that there was an ASF outbreak here. ……the pigs in the pigpen should be culled and then treated innocuously. The television has broadcasted this repeatedly. Almost all the villagers are talking about this. …… Hey, that’s pigs that we raised for such a long time and some of them may not be affected. Of course, we can’t bear it (referring to the culling), but I have to. Once a pig was found to be infected with ASF, all the pigs within three kilometers of the affected pig must be culled to prevent further dissemination. Besides, if my neighbors who raised pigs have already culled theirs, I think there is no other reason to hold mine. I do not like to be portrayed as a selfish person who do not care about other’s interest ……This is a natural disaster. Local government is helping us out of the disaster. Only can we cooperate with them, the loss could be minimized.*

(LY09)

### 4.3. Two Approaches for Farmers to React towards Epidemic

In response to an ASF epidemic, pig farmers finally formed two typical corresponding patterns towards the epidemic, one of problem-orientedness and the other of emotion-orientedness through the stimulation of the epidemic situation and an evaluation process of the epidemic response behavior. In the prevention and control of an ASF epidemic, the problem-orientedness response behaviors include compliance with culling (cooperation, non-cooperation), disinfection (disinfection, non-disinfection), epidemic reporting behavior (reporting, non-reporting), and handling sick/dead pigs (hazardous treatment, disposal without treatment, sale). Pig farmers’ emotional-orientedness responsive behaviors include emotional vetting and emotional support seeking. Pig farmers’ emotions change dynamically over time. They seek emotional support due to their lack of understanding and dissatisfaction with the government’s epidemic prevention policies, such as seeking explanations and seeking compensation.

*Why pigs have not been infected must be culled? I think (the government) should explain it to us. Culling pigs infected is Ok, but we have to cull pigs not infected. They should be tested at least* *. If they were infected, it was not too late to cull them. We have had swine fever before, but only infected pigs were culled. They (refer to the local government) should test it before kill it aimlessly.*

(LY14)

*They (refer to the local government) told us not to slaughter our own pigs the day before, and the next day (they) came to cull the pigs. Nobody asked us... Regardless of the size of the pig, the compensation for farmers is 1200 yuan. I have nine pigs, all of them over three hundred kilograms. If I sold ten yuan a kilo, my pigs worth 3000 yuan each. My neighbor has three little pigs, less than one hundred kilograms each. If sold the same price, they worth less than 1000 yuan. They earn a lot! This is unfair! That’s why I think our compensation should vary according to the size.*

(TY04)

Some of pig farmers mainly complained for the economic loss because of ASF, which symbolized as panic and helplessness. For example, a pig farmer who lost the family income expressed:

*Pigs are the major income of our family. Because of ASF, all pigs have been culled now. I don’t know what to do and all of sudden my pigs are gone. It is not easy to find alternative jobs, and I am desperate.*

(TJ06)

## 5. Conclusions

### 5.1. Principle Findings

Taking ASF epidemic as an example, this study explored the underlying mechanism of pig farmers’ coping behaviors by using a grounded theory approach. The major conclusion and contributions are summarized as follows.

For the epidemic scenario, firstly, emergency response, information sources, and information channels are the basis for risk perception and behavioral perception by pig farmers. The complementary information network of traditional and new media that has been formed in rural China included face-to-face propaganda, epidemic prevention services provided by grassroots government staff, timely announcement of information by village radio and bulletin boards, as well as new media channels such as WeChat and TikTok. Under this condition, pig farmers were able to obtain ASF-related information and make correct behavioral decisions quickly after an ASF outbreak. Secondly, government characteristics and pig farmers’ characteristics have a moderating effect on pig farmers’ epidemic prevention behavioral decisions. Individual pig farmers’ epidemic prevention behavior is affected by various factors such as the strength of government policy implementation, economic interests, social responsibility, and *face (social pressure)*. Among them, social responsibility plays a critical role in pig farmers’ decision-making. This may imply that pig farmers develop a more comprehensive understanding of government policy responding to epidemic compared to the older days.

For the individual decision-making process, firstly, our study demonstrated a strong linkage between the pig farmers’ epidemic risk perception and their behavioral decisions, which is consistent with previous research [20,25]. Secondly, although economic factors are still important factors influencing the decision of Chinese pig farmers’ epidemic response behavior, it is the information factor that plays a decisive role, which is different from other countries such as Uganda [18], the Netherlands [20], and Belgium [21]. In these countries, pig farmers’ epidemic response behavior is mainly influenced by economic factors. A possible explanation could be that with the vast increase of household income among Chinese rural residents, pig farmers give greater weight to other non-economic factors, such as public interest and health in their epidemic prevention behavior decisions. In this vein, personal economic losses are not that important in this case. Finally, the perceived cost of being punished is also an important influencing factor in pig farmers’ decision-making. Among them, external penalties come from strict government enforcement, which is consistent with previous research [27]. On the other hand, the internal punishment comes from the sense of moral responsibility and social pressure. The decision of pig farmers’ epidemic prevention behavior is embedded in specific cultural traditions in rural areas, for example, the *acquaintance society* structure in rural areas has a strong inhibiting effect on pig farmers’ non-cooperative behaviors. Meanwhile, pig farmers are concerned about losing face in case they would be reported by their acquaintances, and thus give up committing violations. The above influencing factors provide a theoretical basis for understanding the differences in farmers’ epidemic response behaviors and guiding their response behaviors.

Thirdly, we have distinguished patterns of pig farmers’ coping behavior in the event of an epidemic, in terms of the behavior of epidemic response. Our study has classified pig farmers’ epidemic coping behavior into two approaches, one of problem-orientedness and the other of emotion-orientedness. Their differentiations in coping behaviors were closely related to their perception of epidemic risk and their attitude towards coping behaviors. In general, the higher the perception of epidemic risk, the more likely their adoption of problem-oriented coping behaviors. If farmers possess a higher behavioral efficacy and a comparatively lower perception of behavioral cost, they are more likely to adopt corresponding coping behaviors, and vice versa.

### 5.2. Practical Implications

It is crucial to facilitate rational epidemic coping behaviors of farmers rapidly since their participation affects the effectiveness of the overall epidemic prevention and control performance. Our research has the following practical implications. First, the government is expected to initiate effective health campaigns to make farmers form a reasonable risk perception. Before the epidemic, local governments should develop a comprehensive risk communication scheme and raise farmers’ awareness of potential epidemic through various communication channels constantly [39]. The content of the risk communication materials should be simple, correct, and accompanied by pictures for farmers to understand and develop a reasonable risk perception [40,41]. Once the epidemic happens, the government must release relevant information to farmers via various media channels, the internet, in particular, properly guide online public opinion, and help farmers to understand the current situation [42,43]. Meanwhile, the government should also attach a great importance to the face-to-face communication with farmers right after the epidemic, which is effective in raising farmers’ awareness of their responsibility in epidemic prevention and control. In addition, rapid emergency response can reduce farmers’ epidemic risk perception and lead to more compliance in epidemic response behaviors. Our research indicates that both government emergency response capability and government characteristics affect farmers’ risk perception and their willingness of epidemic responsive behaviors. It is highly recommended that the government take effective emergency responsive measures to reduce farmers’ property losses and secure food safety.

Secondly, the adjustment of farmers’ perception of responsive behavioral cost and behavioral efficacy is likely to facilitate rational response behaviors. Our research implies that when farmers perceive a high behavioral cost to response, they are more likely to vet their emotions and take measures against the government epidemic prevention if they were desperate [44]. Correspondingly, we propose the amendment of compensation policy for culling breeding animals to further decrease farmers’ epidemic behavioral cost. For example, the compensation standard cannot be one-size-fit-all; instead, it needs to be refined taking into consideration the breed and size of the pig, as well as the pork price. In addition, various measurements are required to improve public trust in government. For instance, they include exerting harsh punishment for offenders, strengthening the supervision of the compensation payment, standardizing the payment procedures, and improving the transparency of compensation information.

### 5.3. Limitations

This paper has limitations that imply future research opportunities. First, this paper explains the underlying mechanism of farmers’ epidemic response behavior from the evaluation of epidemic prevention and control process, and future research could also consider other influencing factors, such as farmers’ prior experience of epidemic situations and the variations of local countermeasures deployed. Secondly, this paper highlights two moderating variables from both the perspectives of government and farmers during the evaluation of epidemic prevention and control process. However, it is still possible that other variables will facilitate or hinder farmer’s responsive behaviors, for example, the participation of non-government organizations and changing conditions of ASF outbreaks. Finally, although this paper aimed to reveal what determines farmers’ epidemic response behaviors, it failed to further elaborate the strength of the relationship across variables. Future research could also involve conducting large-scale questionnaire surveys to quantify the relationships between major variables, as well as to improve the applicability of the results.

## Figures and Tables

**Table 1:** The background information of the participants.

Categories		Number of Participants	Percentage (%)
Gender	Male	31	68.9
	Female	14	31.1
Age	Under 40 years old	9	20.0
	40–50 years old	10	22.2
	Over 50 years old	26	57.8
Breeding experience	Less than five years	12	26.7
	5–10 years	15	33.3
	Over 10 years	18	40.0
Breeding size	Under 30 pigs	32	71.1
	30–100 pigs	11	24.5
	Over 100 pigs	2	4.4
Education	Junior high school or below	21	46.7
	High school	18	40
	Junior college	6	13.3

**Table 2:** Preliminary categories formed in open coding.

Concept	Preliminary Categories
Isolation, quarantine, culling, sanitization, disinfection, propaganda	Government Response
cooperation with the government’s quarantine and disinfection measures, do not consume pork, and monitor prevention and control behaviors	Public Response
Government or public emergency response speed after an ASF outbreak: rapid response, slow response	Response speed
Government as a source of information about ASF: central government, provincial government, city government, county government, town government, village committee	Government
Unofficial media release information related to ASF: unofficial websites, self-published media	Media
People who spread information about African swine fever: neighbors, relatives, friends	Public
Information dissemination through paper-based printed materials: newspapers, bulletin boards	Paper-based media
Information dissemination through electronic technology and equipment channels: internet, TV, telephone, radio	Electronic media
Face-to-face transmission of information: verbal transmission, door-to-door service	Face-to-face communication
Strength of grassroots government in implementing epidemic prevention and control policies: strong, weak	Strength of policy implementation
Government resource investment in epidemic prevention and control: large, small	Resource investment
Government credibility: high, low	Government credibility
Breeding size of farmers: free-range farmers (<30 heads), large-scale farmers (>=30 heads)	Breeding size
Breeding income share of total household income: 0–30%, 31–60%, 61–100%	Breeding income share
Sense of social responsibility of farmers: high, low	Sense of social responsibility
Farmers’ perception of the probability of ASF epidemic: large, average, small	Probability of epidemic
Farmers’ perception of the severity of the consequences of the ASF epidemic: serious, average, not serious	Severity of consequences
Effectiveness and benefits of adopting response behaviors: assessment of one’s own behavioral ability to prevent disease, assessment of the effectiveness of individual disease prevention on overall disease prevention	Behavioral efficacy
Costs or losses of adopting coping behaviors: monetary costs, energy costs, time costs, re-employment costs	Behavioral cost
Farmers simply process information based on intuition: direct action	Heuristic path
Farmers process information after searching for it and analyzing it rationally: information search, search for response behavior options, analysis of response behavior options	Analytical path
Behaviors related to cooperative culling: cooperative, uncooperative	Cooperative culling behaviors
Behavior related to disinfection: disinfection, no disinfection	Disinfection behavior
Behavior related to epidemic reporting: reporting, not reporting	Epidemic reporting behavior
Behavior related to disposal of dead pigs: disposal without harm, disposal without disposal, sale	Handling behavior of dead pigs
Emotional distress relief through emotional catharsis: doubts, dissatisfaction, complaints, anger, panic, helplessness	Emotional catharsis
Seeking social support to relieve emotional distress: seeking explanation, seeking comfort, seeking compensation	Seeking emotional support

**Table 3:** Main categories formed by axial coding.

Preliminary Category	Main Category
government response, public response, response speed	emergency response
government, the media, the public	information sources
print media, electronic media, face-to-face communication	access to information
policy implementation strength, resource input, government credibility	governmental characters
breeding scale, ratio of breeding on household income, social responsibility	the farmer’s characters
epidemic incidence probability, severity of consequences	epidemic risk perception
behavior efficiency, behavior cost	coping behavior perception
heuristic path, analytical path	information processing
cooperation with culling behavior, disinfection behavior, epidemic report behavior, disease dead pig disposal behavior	problem-orientedness
empathy and seeking emotional support	emotion-orientedness

## Data Availability

The data are available upon request of the corresponding author.

## Appendix A. Interview Outline

**Section 1**: Background Information of the pig farmers, including their gender, age, educational level, breeding size, breeding experience, household income structure.

**Section 2**: Pig farmers’ risk perception, attitude and coping strategies towards ASF.

1. Which channels did you get information on the ASF epidemic from?
2. Do you know the source, etiology and symptoms of ASF?
3. What has the government taken to prevent and control ASF?
4. How do you say about the government measures?
5. How do you perceive the ASF epidemic?
6. How has the ASF epidemic affected you and your family?
7. What actions have you taken to deal with the ASF outbreak?
8. What are your reasons for taking this action?
